# Disrupted Resting-State Default Mode Network in Betel Quid-Dependent Individuals

**DOI:** 10.3389/fpsyg.2017.00084

**Published:** 2017-01-30

**Authors:** Xueling Zhu, Qiuling Zhu, Canhua Jiang, Huaizhen Shen, Furong Wang, Weihua Liao, Fulai Yuan

**Affiliations:** ^1^Health Management Center, Xiangya Hospital, Central South UniversityChangsha, China; ^2^School of Humanities and Social Sciences, National University of Defense TechnologyChangsha, China; ^3^Obstetrics Department, Jinan Maternity and Child Care HospitalJinan, China; ^4^Department of Oral and Maxillofacial Surgery, Xiangya Hospital, Central South UniversityChangsha, China; ^5^Department of Radiology, Xiangya Hospital, Central South UniversityChangsha, China

**Keywords:** betel quid dependence, default mode network, independent component analysis, resting-state, fMRI, functional connectivity

## Abstract

Recent studies have shown that substance dependence (addiction) is accompanied with altered activity patterns of the default mode network (DMN). However, the neural correlates of the resting-state DMN and betel quid dependence (BQD)-related physiopathological characteristics still remain unclear. Resting-state functional magnetic resonance imaging images were obtained from 26 BQD individuals and 28 matched healthy control subjects. Group independent component analysis was performed to analyze the resting state images into spatially independent components. Gray matter volume was examined as covariate with voxel-based morphometry to rule out its effect on the functional results. The severity of BQD was assessed by the BQD Scale (BQDS). We observed decreased functional connectivity in anterior part of the DMN including ventral medial prefrontal cortex, orbital MPFC (OMPFC)/anterior cingulate cortex (ACC). Furthermore, the functional connectivity within the OMPFC/ACC in BQD individuals was negatively correlated with BQDS (*p* = 0.01, *r* = -0.49). We reported decreased functional connectivity within anterior part of the DMN in BQD individuals, which provides new evidence for the role of the DMN in the pathophysiology of BQD.

## Introduction

Betel quid (BQ) is the fourth most commonly consumed psychoactive substance in the world, following only alcohol, nicotine, and caffeine (Boucher and Mannan, 2002; Warnakulasuriya and Peters, 2002). Although BQ is chewed by approximately 600 million people globally, its use is concentrated in South Asia, Southeast Asia, and Pacific islands (Gupta and Warnakulasuriya, 2002). The composition and method of BQ chewing can vary widely from country to country. In Hunan in the Mainland China, BQ is consumed a halved dried husk of the areca fruit (not the solid areca nut) marinated with bittern (containing lime) and diverse flavored additives (Lee et al., 2014). From a public health perspective, BQ chewing is associated with a variety of health issues, most notably oral cancer and precancerous conditions such as leukoplakia and oral submucous fibrosis (Trivedy et al., 2002). Consequently, BQ has been classified as a Group 1 carcinogen by the International Agency for Research on Cancer (IARC Working Group on the Evaluation of Carcinogenic Risks to Humans, 2004; Lin et al., 2006).

Despite the global pervasiveness of BQ, the initial research literature on BQ mostly focused on the epidemiological and biological aspects of BQ chewing (Ghani et al., 2011; Lee C.H. et al., 2012). In recent years, some studies have focused on the behavioral and psychological aspects of BQ chewing. Due to the lack of corresponding criteria for the BQD in the DSM-IV, the instruments to assess BQ dependence syndrome varied from the Diagnostic and Statistical Manual of Mental Disorders-IV (DSM-IV; American Psychiatric Association (APA), 2000; Benegal et al., 2008), the International Classification of Diseases-10 (World Health Organization, 1992; Mubeen et al., 2010) to some dependence scales for other substances such as opioids (Winstock, 2002) or tobacco (Bhat et al., 2010). Recently, based on previous research findings and the diagnostic criteria of Substance Dependence in DSM-IV, the Betel Quid Dependence Scale (BQDS) has been developed to specially measure BQ dependence, which is more suitable for Chinese-speaking chewers and valid for current English-speaking male and female chewers in Guam (Lee C.Y. et al., 2012; Herzog et al., 2014). The BQDS has been gradually approved in Chinese samples (Liu et al., 2016a,b).

To date, few studies have been made in understanding neurophysiological aspects of BQ chewing. Structural imaging studies revealed abnormal gray matter volume in bilateral dorsolateral prefrontal cortex (PFC), anterior cingulate cortex (ACC), and mid-brain in BQD individuals (Chen et al., 2015). With the rapid progress of neuroimaging techniques, resting-state functional magnetic resonance imaging (fMRI) has been regarded as a useful tool to investigate brain activity (Fox and Raichle, 2007). By use of the amplitude of low-frequency fluctuation (ALFF) and regional homogeneity (ReHo) analysis, Liu et al. (2016a) found that BQD individuals with decreased spontaneous cerebral activity in the prefrontal gyrus and increased spontaneous cerebral activity in the primary motor cortex area, temporal lobe as well as some regions of occipital lobe. Using the seed-based functional connectivity method, the BQD group showed increased connectivity from ACC to pons, caudate, thalamus, midbrain and cerebellum, and decreased connectivity from ACC to medial PFC, parahippocampal/hypothalamus and precuneus (Liu et al., 2016b). The BQD-related structural and functional abnormalities suggested the dysfunction of the reward system, cognitive system, and emotion system in BQD individuals, which showed the similar neurological disruption as the other addiction (Volkow et al., 2012). However, the neural mechanism underlying BQD still remains largely unclear, and further investigation is needed.

Though the brain regions related to the reward, memory and execution function have received great attention for addicted individuals, there is also evidence of a significant disruption in self-awareness in addiction, which includes impaired awareness of disease, his/her need for treatment, and/or his/her strong desire for the drug (Goldstein et al., 2009b; Volkow et al., 2012). The neurocircuitry underlying self-awareness in addiction is still poorly understood. According to the models of addiction, neuroimaging studies have implicated recruitment of several cortical and subcortical midline brain regions, such as MPFC, ACC and hippocampus (Kosten et al., 2006; Goldstein et al., 2009a; McClernon et al., 2009; Seo et al., 2013), which are crucial components of the so-called default mode network (DMN) in relation to resting-state brain function (Raichle et al., 2001; Greicius et al., 2003). The DMN is a collection of brain regions, which reliably deactivate during goal-directed behaviors and is more active at baseline (Fox et al., 2005). The DMN is suggested to be involved in self-referential processes such as the process of internal states (Northoff et al., 2006; Andrews-Hanna et al., 2014). Considering that the disruption self-awareness is pervasive in addiction, it can be hypothesized that the DMN might play an important role in the physiopathology of addiction (Goldstein et al., 2009b). Therefore investigating the relationship between the DMN in addiction and addiction-related pathopsychological characteristics is of great interest.

To date, few studies have directly examined addicted-related changes in resting-state DMN activity and those that have been done reported inconsistent functional connectivity patterns. Using independent-components analysis, Ma et al. (2011) found increased functional connectivity in the right hippocampus and decreased functional connectivity in right dorsal ACC and left caudate in the DMN of heroin users. Another study reported heroin-dependent individuals had decreased functional connectivity in several brain regions within the DMN, including the orbital frontal cortex, the bilateral inferior parietal lobe, the bilateral superior frontal gyrus, the bilateral paracentral lobule, the left PHG and the right middle temporal gyrus (Ma et al., 2015). As reported that the DMN is not a unitary system but rather is composed of smaller and distinct subsystems, Li et al. (2016) found decreased functional connectivity in anterior subnetwork of the DMN in heroin addicts. Wang et al. (2016) further revealed a decreased correlation between the DMN and visual networks and task-positive networks in heroin addiction. What mentioned above implies the significance of the DMN in understanding the pathophysiology of addiction. However, the BQD-related DMN activity still remains unclear.

To our knowledge, there is no study examining the resting-state DMN connectivity in BQD individuals. The first goal of this current study was to investigate the altered FC pattern of DMN underlying BQD psychopathology. Additionally, gray matter volume of all participants was calculated to be covariates to clarify that altered connectivity are independent of volume change (Oakes et al., 2007; Liu et al., 2016a). Our second goal was to examine the association of altered resting-state DMN connectivity with the severity of BQD in BQD individuals. Given the role of anterior DMN in addiction (Li et al., 2016), we hypothesized that BQD subjects would also exhibit decreased functional connectivity in anterior part of the DMN, which would negatively correlate with BQD severity.

## Materials and Methods

### Participants

26 BQD subjects were recruited from the outpatient department of stomatology at Xiangya Hospital of Central South University in Changsha, Hunan, China. The BQD subjects met the DSM-IV criteria for substance use disorders determined from the Structured Clinical Interview. A licensed psychiatrist, at MD level, conducted all clinical interviews. Persons without use of BQ or areca nut were defined as “healthy subjects.” 28 healthy control subjects were recruited through a combination of targeted site sampling, advertisement and snowball sampling referrals. Participants were excluded if they (i) met criteria for other substance dependence at any time; (ii) were minority rather than Han Chinese; (iii) had a medical condition or disease with likely significant central nervous system effects; (iv) had a history of head injury with skull fracture or loss of consciousness of greater than 10 min; (v) had a physical problem that would render study measures difficult or impossible; (vi) had any current or previous psychiatric disorder; (vii) had a family history of psychotic disorder; (viii) had undergone current or previous use of electroconvulsive therapy; or (ix) contraindications for MRI scanning. The study protocol was approved by Xiangya Hospital of Central South University of Hunan Province, Changsha, China. All participants were aware of the purpose of the study and signed an informed consent before the study. The participants received a small financial compensation for their participation.

Although alcohol and smoking dependency have been ruled out before recruitment, the frequency and quantity of alcohol and smoking consumption for all participants were also assessed, because both wine and cigarette play an important role in Chinese life. The smoking status is recorded by the average number of the cigarettes smoked every day. The alcohol status is defined as the average grams of alcohol consumed every month. In order to reduce the influence of alcohol, the alcohol status of all participants recruited in this study is occasional, which is defined as once or twice every month.

### Measures

#### BQ Dependence

The severity of BQ dependence was assessed by the BQDS, which is designed according to the diagnostic criteria of “Substance Dependence” from DSM-IV (Lee C.Y. et al., 2012). When the individuals were considered as BQ dependence, the commonest criterion endorsed was spending a great deal of time followed by tolerance, use despite harm, using more than intended and withdrawal. As a 16-item self-report instrument, the BQDS comprises of three factors: “physical and psychological urgent need,” “increasing dose,” and “maladaptive use.” The BQDS had an optimal cut-off score of 4, the optimal sensitivity was 0.926 and the specificity was 0.977, with the predictive accuracy up to 99.3%. The BQDS was found to have good internal consistency (α = 0.92) and construct validity, which exhibited high degrees of reliability and validity in both the English-speaking and Chinese-speaking chewers (Lee C.Y. et al., 2012; Herzog et al., 2014).

### Data Acquisition

Images were obtained using a Siemens Skyra 3T scanner with a standard head coil. Participants wore a standard head coil fitted with foam padding to minimize head movement and diminish scanner noise. During scanning, all participants were required to remain motionless, keep their eyes closed and try not to systematically think of anything. After scanning, the participants were asked about their statement during scanning.

Resting-state fMRI images were acquired with a single-shot, gradient-recalled echo-planar imaging sequence oriented parallel to the line of the anterior-posterior commissure. The following parameters were applied: repetition time = 2000 ms, echo time = 30 ms, flip angle = 80°, field of view (FOV) = 240 mm × 240 mm, matrix = 64 × 64, slice thickness = 4 mm, slice gap = 1 mm, number of slices = 32. For each participant, 216 volumes were obtained, and the scan lasted 432s.

High-resolution 3-dimensional (3D) structural images were acquired using a T1-weighted, magnetization-prepared rapid gradient-echo sequence. The following parameters were applied: repetition time = 1900 ms, echo time = 2.01 ms, flip angle = 9°, FOV = 256 mm × 256 mm, matrix = 256 × 256, slice thickness = 1 mm, slice gap = 0 mm, and number of slices = 176.

### Data Preprocessing

We used the voxel-based morphology technique (VBM)^1^ to investigate gray matter volume over the whole brain in BQD. VBM analysis was conducted using the statistical parametric mapping software package (SPM8^2^). The images were segmented into gray matter, white matter and cerebrospinal fluid using a unified segmentation approach (Ashburner and Friston, 2005). Then, the gray matter partitions of each subject in the native space were high dimensionally registered and normalized to the standard Montreal Neurological Institute space using diffeomorphic anatomical registration through exponentiated lie algebra (DARTEL) normalization as implemented in the SPM8. After normalization, the gray matter images with modulation were smoothed with a Gaussian filter of 8 mm full-width half-maximum kernel. The resultant images were used for statistical analyses.

Resting state fMRI images were performed with Data Processing Assistant for Resting-State fMRI (DPARSF) professional software^3^ (Chao-Gan and Yu-Feng, 2010). For each individual participant, the first 10 functional images were excluded from analysis. Subsequent images were corrected by slice timing and realigned for head motion. One BQD and one healthy subject were excluded because their translation or rotation exceeded ± 1.5 mm ± 1.5°. The individual T1-weighted structural images were coregistered to functional images. The transformed structural images were then segmented into gray matter, white matter, and cerebrospinal fluid (CSF) and normalized to Montreal Neurological Institute (MNI) space. These transformation parameters were also applied to the functional images. The normalized functional images were resampled at a resolution of 3 mm × 3 mm × 3 mm and spatially smoothed with a 6-mm full width at half maximum Gaussian kernel. The sources of spurious variance were regressed out including six parameters from head-motion correction (Friston 24-parameter model), white matter and CSF signal. Finally, functional images with linear trend were removed by temporal bandpass filtering (0.01–0.08 Hz). At last, data from 25 BQD and 27 healthy control subjects was included into the next analysis.

### Independent Component Analysis and Identification of DMNs

Spatial independent component analysis (ICA) was conducted for 52 participants using the Group ICA of fMRI Toolbox (GIFT) software (Medical Image Analysis Lab, University of New Mexico, New Mexico^4^), which has been widely used to identify and quantify distributed patterns or spatial networks of correlated activity (Zhu et al., 2012). There were three main steps of group ICA: data reduction, independent component (IC) separation, and back reconstruction. The number of ICs was set 20 for ICA separation according to previous resting-state studies (Ma et al., 2011; Li et al., 2013). After ICA separation, a template of the DMN was used to select the greatest best-fit component for each subject. The standard DMN template was from a meta-analytic modeling provided by Angela R. Laird, Ph.D. (Research Imaging Institute, University of Texas Health Science Center, San Antonio, TX, USA; Laird et al., 2009). Then, a multiple regression was performed over voxels and components that best fit the default mode template were selected.

### Statistical Analysis of the DMN

After being extracted from all subjects, the best-fit components representing the DMN were gathered in each group separately using the one-sample *t*-test. Thresholds were set at *p* < 0.05 (false discovery rate [FDR] correction). Subsequently, the two-sample *t*-tests were used to compare the best-fit components between two groups (*p* < 0.05 with FDR correction). In order to exclude the possible effect on the final results, the gray matter volume was introduced as covariate in the two-sample *t*-tests, as well as age, gender, years of education, smoking, and alcohol status. The group comparisons were restricted (masked) to the voxels within the corresponding DMN. The mask was created by uniting the DMN regions of the BQD and the control subjects, which were obtained from the one-sample *t*-test results (*p* < 0.05 with FDR correction).

### Correlation Analysis

To investigate the association between the activity of the DMN and demographic and clinical characteristics, Spearman correlation was calculated. The regions showing significantly altered functional connectivity between the BQD and control groups were extracted as regions of interest. Correlation analysis was implemented to the mean value of the functional connectivity in regions of interest and age, years of education, BQDS, duration of BQ, dosage of BQ, smoking, and alcohol status for using SPSS 22.0 (IBM SPSS Inc., USA).

## Results

### Demographics and Clinical Characteristics

The demographic and clinical characteristics for BQD and healthy control participants were shown in **Table 1**. The BQD individuals exhibited a mean BQDS of 10.92 ± 1.66, a mean duration of BQ 13.20 ± 5.31 years and average dosage of BQ 48.80 ± 17.22 g daily. The BQD and control groups did not differ significantly in terms of age, gender, education, smoking and alcohol status (*p* > 0.05).

**Table 1:** Demographic and clinical characteristics of participants.

Characteristics	BQD	Control	*t*/χ^2^	*p*
Age (mean ± SD years)	30.28 ± 5.26	28.30 ± 5.82	-1.29^a^	0.21
Gender (female/male)	6/19	9/18	0.55^b^	0.46
Education (years)	14.08 ± 5.11	14.30 ± 2.73	0.99^a^	0.32
BQDS	10.92 ± 1.66	NA		
Duration of BQ (years)	13.20 ± 5.31	NA		
Dosage of BQ (g/day)	48.80 ± 17.22	NA		
Smoking status (c/day)	18.40 ± 5.72	17.20 ± 4.35	0.84^a^	0.41
Alcohol status (g/month)	5.16 ± 2.08	5.27 ± 1.97	0.83^a^	0.40

*BQ, betel quid; BQD, betel quid dependence; BQDS, betel quid dependence scale; c, cigarette; g, gram; a, independent-samples *t*-test; b, Chi square test.*

### Statistical Comparison of DMNs

**FIGURE 1:**
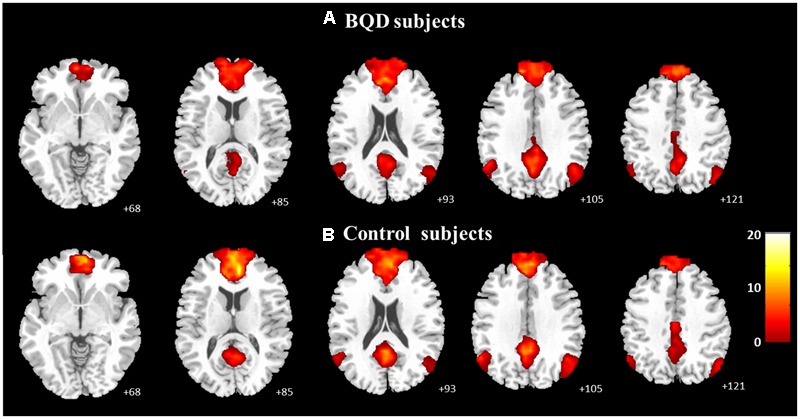
**Axial images showing the group default-mode network extracted by independent component analyses in BQD (A**; *n* = 25, *p* < 0.05 with FDR correction) and in healthy control subjects (**B**; *n* = 27, *p* < 0.05 with FDR correction).

The one-sample *t*-tests (*p* < 0.05 with FDR correction) revealed the respective spatial pattern of the DMN in the BQD and healthy control subjects (**Figures 1A,B**). Increased functional connectivity was identified in the MPFC, PCC/precuneus, bilateral angular gyrus, inferior temporal cortex, and medial temporal lobes in both groups, which correspond to the typical distribution of the DMN (Raichle et al., 2001).

**FIGURE 2:**
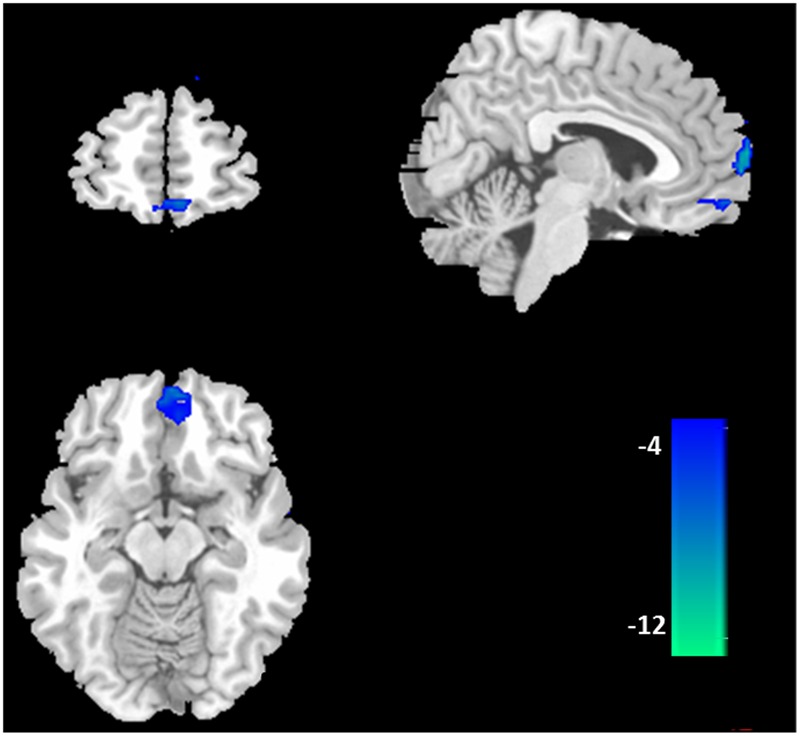
**Difference in functional connectivity of the default mode network between the BQD and healthy control subjects.** According to the results of two-sample *t*-tests, the BQD subjects showed significantly decreased functional connectivity in ventral medial prefrontal cortex, orbital MPFC/anterior cingulate cortex (*p* < 0.05 with FDR correction).

Furthermore, the two-sample *t*-tests (*p* < 0.05 with FDR correction) showed that there were significant differences between the DMNs of the two groups. Relative to healthy control subjects, the BQD individuals showed decreased resting functional connectivity in anterior part of the DMN including ventral MPFC (VMPFC), orbital MPFC (OMPFC)/ACC (**Figure 2**; **Table 2**).

**Table 2:** Brain areas with significantly decreased DMN activation in BQD compared with control subjects.

Brain regions	BA	x, y, z^a^	*T*	voxels
VMPFC	10	3 63 -3	-12.90	69
OMPFC/ACC	11/32	-3 57 -15	-8.11	63

*VMPFC, ventral medial frontal cortex; OMPFC, orbital medial frontal cortex; ACC, anterior cingulate gyrus; BA, brodmann; a, Montreal Neurological Institute coordinates.*

### Correlation Analysis

**FIGURE 3:**
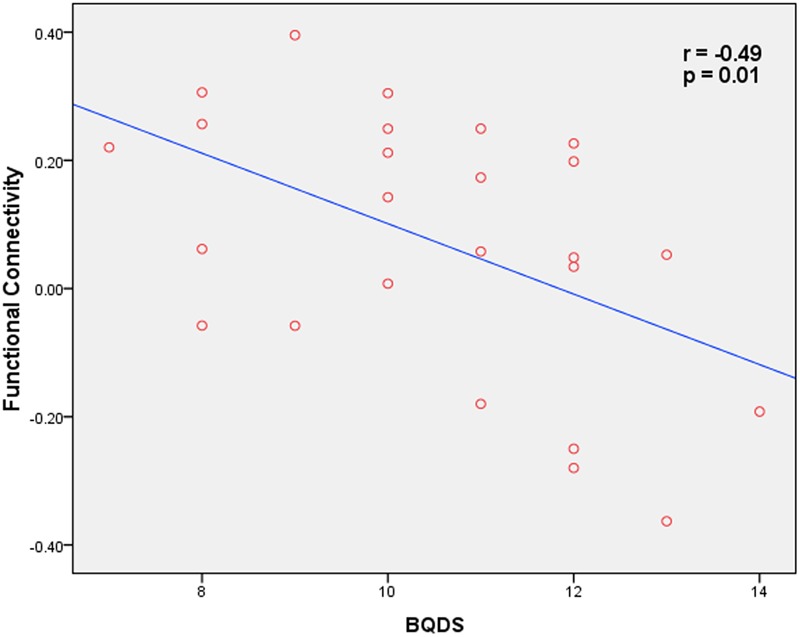
**Correlation map between functional connectivity within the OMPFC/ACC and BQD scores in BOQ subjects (*p* = 0.01, *r* = -0.49)**.

Correlation analysis revealed that functional connectivity in OMPFC/ACC showed a significant negative correlation with BQDS in BQD individuals (*p* = 0.01, *r* = -0.49; **Figure 3**). In addition, no significant correlation was found between functional connectivity within the DMN and the other clinical variables (age, years of education, duration of BQ, dosage of BQ, smoking and alcohol status).

## Discussion

Using ICA methodology, the present study demonstrated altered resting-state DMN functional connectivity in BQD individuals. As we hypothesized, the BQD individuals exhibited decreased functional connectivity in the VMPFC and OMFC/ACC within anterior part of the DMN. Furthermore, the BQDS negatively correlated with functional connectivity in the brain regions of OMPFC/ACC. To our knowledge, our study is the first to investigate the relationship between the DMN and BQD-related characteristics, which demonstrate the presence of aberrant DMN activities in BQD individuals.

Compared with healthy control subjects, the BQD individuals showed decreased resting-state functional connectivity in VMPFC and OMPFC/ACC, which all located in PFC parts of the DMN. As an important part of the DMN, the PFC is involved in both emotional and cognitive functions (Maddock, 1999). The PFC has wide links with both affective-limbic areas (such as the amygdala, hippocampus, and hypothalamus) and executive control and emotional processing areas (such as OFC and ACC; Maddock, 1999). Accumulating evidence has demonstrated the prominent role of the PFC in addiction (Volkow and Fowler, 2000; Volkow et al., 2012), which include self-control to terminate actions that are not advantageous to the individual, salience attribution and maintenance of motivational arousal that is necessary to engage in goal-driven behaviors, and self-awareness (Volkow and Fowler, 2000; Goldstein and Volkow, 2011). Disruption of the PFC in addiction underlies not only compulsive drug taking but also accounts for disadvantageous behaviors that are associated with addiction and the erosion of free will (Goldstein and Volkow, 2002, 2011; Hornak et al., 2004; Volkow et al., 2005, 2007). Consistent with previous studies of addiction (Ma et al., 2011; Li et al., 2016), our study provides new evidence for abnormal PFC functional connectivity in BQD individual, which suggests similar mechanism of the DMN activity in addiction.

The current study showed decreased functional connectivity in VMPFC and OMPFC/ACC in BQD individuals relative to healthy group, which is consistent with the model of addiction circuitry (Koob and Volkow, 2010; Volkow et al., 2012). The VMPFC and OMPFC were reported to be involved in salience attribution and goal-directed behaviors as well as the ACC in inhibitory control and awareness (Volkow et al., 1993, 2001; Hommer, 1999). A growing body of literature has showed that VMPFC, OMPFC, and ACC as key structures involved in cocaine-addicted (Volkow et al., 1993, 2005; Franklin et al., 2002; Bolla et al., 2003), methamphetamine-addicted (Volkow et al., 2001; Sekine et al., 2003), heroin-addicted (Sell et al., 2000), and marijuana-addicted subjects (Volkow et al., 1996). Improper modulation of these regions in addicted subjects has been suggested to underlie the enhanced incentive motivational value of drugs and the users’ loss of control over drug intake (Volkow and Fowler, 2000). In BQD individuals, decreased resting state spontaneous cerebral activity in MPFC and OFC was reported by Liu et al. (2016a). From a new perspective of brain functional network, our study reveals that BQD individuals showed decreased functional connectivity in VMPFC and OMPFC/ACC, which implies the disrupted network-level functional integration in these brain areas.

It is worth noting that the resting state functional connectivity in OMPFC/ACC was negatively correlated with BQD scores in BQD individuals. Previous resting state studies have identified the correlation between OMPFC/ACC and BQD. For example, Liu et al. (2016a) certificated that the BQD scores were negatively related to brain spontaneous cerebral activity in the ACC (Liu et al., 2016a). Additionally, an increasing number of neuroimaging studies have indicated that addicted subjects exhibited a reduction in striatal dopamine D2 receptor (Koob, 1992; Heinz et al., 2004), which are associated with reduced activity of the orbitofrontal cortex including OMPFC and ACC, resulting in the deregulation of frontal regions by dopamine in deficits in response inhibition and impulse control in addiction (Volkow et al., 2007). Consistent with these prior findings, our results provide new evidence for dysfunction in OMPFC/ACC in BQD and further suggest such dysfunction may serve as a specific biomarker during BQD development.

There are several other potential methodological limitations to the interpretation of the results. First of all, the causal relationship between changes in functional connectivity of the DMN and BQ use cannot be fully determined in current design, because our study can only be observed as a cross-sectional study. Secondly, the proportion of females in the sample was relatively small because of the reality of few BQD women. Thirdly, it is always possible that the results could be confounded by the use of other substances, such as cigarettes and wine, although all the recruited subjects resulted from the strict inclusion and exclusion criteria and there was no significant difference in cigarettes and wine between the BQD and healthy control group. Finally, despite of the important role of the DMN in BQD individuals, future studies are needed to explore the brain circuits involve in reward, memory and executive function.

## Conclusion

Using ICA, we identified decreased functional connectivity within the anterior parts of the resting state DMN in BQD individuals relative to the healthy controls. There was significantly negative correlation between functional connectivity in OMPFC/ACC regions and the severity of BQD. Our results highlight the important role of the DMN in the pathophysiology of addiction and suggest that abnormal DMN activity may be a trait associated with BQD.

## Author Contributions

XZ and FY conceived and designed the experiments. HS, FW, and WL conducted the experiments and collected data. QZ and HS analyzed the results. XZ, QZ, and CJ wrote the main manuscript text. All authors reviewed the manuscript.

## Conflict of Interest Statement

The authors declare that the research was conducted in the absence of any commercial or financial relationships that could be construed as a potential conflict of interest.

## Footnotes

1 http://dbm.neuro.uni-jena.de/vbm8

2 http://www.fil.ion.ucl.ac.uk/spm

3 http://www.restfmri.net

4 http://icatb.sourceforge.net/
